# Developing and Testing a Scale Designed to Measure Perceived Phubbing

**DOI:** 10.3390/ijerph17218152

**Published:** 2020-11-04

**Authors:** Meredith E. David, James A. Roberts

**Affiliations:** Hankamer School of Business, Baylor University, One Bear Place 98007, Waco, TX 76798, USA; jim_roberts@baylor.edu

**Keywords:** phubbing, smartphones, social exclusion, social media, anxiety, depression

## Abstract

Phubbing (phone snubbing) has become a commonplace behavior. The more we are phubbed the more likely we are to phub others. The extraordinary attention-grabbing ability of the smartphone would only be an interesting story if not for its impact on social media use and, ultimately, stress and depression. In Study 1 (n = 258, *M*_age_ = 20), we develop a parsimonious and valid measure of phubbing. Extant “phubbing” measures all lack important qualities needed to be able to assess phubbing with a brief and valid scale that can be replicated and used in a variety of research settings. In Study 2 (n = 157, *M*_age_ = 39), we test and extend the David and Roberts (2017) phubbing model, while further validating our perceived phubbing measure. We use Social Exchange Theory and Kardefelt-Winther’s (2014) model of compensatory internet use as theoretical support for our expected findings. Results find that phubbed individuals experience a sense of social exclusion that, paradoxically, is associated with an increased use of social media. This increased use of social media is associated with higher reported levels of anxiety and depression. Future research directions and study limitations are discussed.

## 1. Introduction

The US, like many other developed countries around the world, is a nation of distracted individuals. A primary culprit of such distraction is the smartphone [1,2,3]. The modern smartphone is ubiquitous [4]. For many users, their smartphone is always within reach even while sleeping [5]. Its multifunctionality makes the smartphone seemingly indispensable [6,7]. In the current highly digitized environment, the use of one’s smartphone in the company of others has become a common occurrence [8,9].

Given the above, smartphones may hold sway even during face-to-face conversations and everyday social interactions [10]. Phubbing is a portmanteau. It is a combination of the words phone and snubbing. To be phubbed is “to be snubbed by someone using their smartphone when in your company” [2] (p. 134). Phubbing is an inevitable occurrence. We have all been phubbed and likely have phubbed others [9]. Bjornsen et al. (2017) found one hundred percent of respondents reported some level of phubbing in their relationships [11]. Research by Chotpitayasunondh and Douglas (2016) found that the more one is phubbed, the more they see the behavior as normal. Reciprocation leads to more phubbing [12].

As humans, our need to belong is paramount and vital to our very existence. We are social animals. Those of us with strong social networks live longer and are happier [13,14]. To be socially excluded is to threaten one’s very existence. Baumeister and Tice (1990) argue that our fear of social exclusion is equivalent to our fear of snakes, the dark, and heights [15].

Despite the importance of social relationships to our longevity and well-being, research suggests that both the quantity and quality of social relationships in industrialized societies has decreased [14,16]. It may be that the more time spent online, the less time there is available for face-to-face interactions [17]. Others’ use of their smartphone while in another’s presence can create a sense of social exclusion in the phubbed individual [18]. Avoiding internal and external distractions and giving your full attention to the person you are with is essential to fostering strong social bonds [18], just as eye contact is an essential component of human connection [9,19].

The role phubbing plays in driving a sense of social exclusion and, ultimately, social media use is the primary focus of the present study. The current study is an extension of research by David and Roberts (2017), which found that, when phubbed, enigmatically, people turn to social media instead of face-to-face interactions to restore their sense of inclusion [18]. This relationship is especially important given the potentially negative outcomes associated with heavy social media use [20,21,22].

## 2. Literature Review

### Phubbing Scales

Our increasingly digitized world often interferes with the ability to have uninterrupted interactions with others [8]. Society’s present preoccupation with smartphones has disrupted the normal patterns of interaction between individuals often with a negative influence on the phubbed individual. One goal of the present research is the development of a valid measure of perceived phubbing. Given the increasing prevalence of phubbing and its potentially negative impact on well-being, stress, depression, and anxiety, it is essential that a parsimonious, valid, and reliable scale of phubbing be available.

Several phubbing scales currently exist. Chotpitayasunondh and Douglas (2018a) constructed the 22-item Generic Scale of Being Phubbed (GSBP) [1]. Several potential shortcomings exist regarding using the GSBP scale in research. First, it is 22 items in length. Given the difficulty of getting respondents to participate in surveys and provide thoughtful responses, shorter scales are needed. Second, the GSBP comprises three factors: (1) perceived norms of being phubbed, (2) feeling ignored by others’ phone use, and (3) interpersonal conflict created by others’ mobile phone use. Factors one and three measure constructs that are distinct from the event of being phubbed (Factor 1—perceived norms; Factor 3—interpersonal conflict). 

A phubbing scale developed by Chotpitayasunondh and Douglas (2016) measured the frequency and duration of phubbing and being phubbed [12]. The ability of respondents to estimate the number of times in a week they were either phubbed or phubbed others is highly suspect. The same concern can be expressed for respondents’ ability to estimate the duration (in minutes) they phubbed or were phubbed daily.

The “phubbing” scales (TDIS and TILES) developed by McDaniel and Coyne (2016) measured the broader category of technology interference and the respondent’s perception of how willing their romantic partner was to allow technology to interfere while they spent time together [23].

Lastly, a phubbing scale created by Karadag and co-authors (2015) resulted in a two-factor scale [24]. Problematically, five items that loaded on the second factor of the scale appear to measure what was labelled “smartphone obsession,” which closely resembled the types of questions that should be asked if the goal was to measure smartphone addiction. The existence of this “smartphone obsession” factor would likely confound results if the model being tested also included a smartphone addiction construct/scale. This is a real possibility, given that smartphone addiction is likely an antecedent of phubbing behavior [12].

The present phubbing scale has been adapted from Roberts and David’s (2016) single-factor partner phubbing scale, which has repeatedly been shown to be a valid, reliable, and parsimonious measure of the perceived extent to which an individual feels phubbed by his or her relationship partner [2,25,26,27,28,29]. The present study offers a measure of perceived phubbing, that is, the extent to which an individual perceives experiencing phubbing in day-to-day life. The measure of perceived phubbing is presented and tested in Study 1. Study 2 then further examines whether the perceived phubbing measure exhibits nomological and predictive validity by using the general measure of phubbing to test a model previously supported in the literature. Specifically, David and Roberts (2017) manipulated phubbing and showed its impact on individuals’ feelings of social exclusion and subsequent need for attention and social media intensity; we test the same model but using our measure of phubbing rather than a manipulation as in David and Roberts’ (2017) research [18].

## 3. David and Roberts (2017) Model

**Figure 1:**
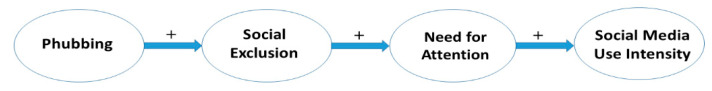
Sequential Mediation Model Tested Based on David and Roberts (2017).

As depicted in Figure 1, David and Roberts (2017) hypothesized that being phubbed leads to a sense of social exclusion [18]. Since the publication of David and Roberts (2017), two studies have found support for the proposed relationship between being phubbed and feelings of social exclusion/ostracism [19,30]. Most of the prior research on phubbing and phubbing-like behavior has investigated its effects amongst romantic partners [2,23,31,32].

Given the importance of feeling connected to our fellow human beings, the sense of exclusion activated by being phubbed increases one’s need to regain inclusion. Being excluded is painful [33]. When we are excluded, parts of our brain that detect and regulate pain are activated. Additionally, our ability to control our thoughts, emotions, and behavior is compromised, as are our abilities to reason and properly perceive time [34]. Once a person feels excluded, his or her paramount concern is to regain a sense of inclusion. To regain a sense of inclusion threatened by a face-to-face interaction, people may turn to their smartphones and social media to engage with others and soothe the pain associated with being phubbed. Kardefelt-Winther’s (2014) theory of compensatory internet use can be used to support the present study’s contention that, when phubbed, people may turn to social media as a means to compensate in response to such a negative life event [35]. According to Kardefelt-Winther’s (2014) theory of compensatory internet use, a phubbed individual may turn to social media to compensate for the perceived phubbing and resultant sense of exclusion [35].

For example, David and Roberts (2017) hypothesized, and found, that this need to feel included led subjects to increasingly turn to social media to regain a sense of inclusion [18]. Individuals are constantly monitoring their levels of inclusion and will redirect their attention resources to seek opportunities to connect when they sense they have been excluded [36]. David and Roberts (2017) extended Leary’s (1990) thinking to suggest that people may move to social media because they feel that reinvesting time in face-to-face interactions may lead to the same exclusion as caused by the phubbing [18,37]. Leary (1990) posits that imagining that you have been excluded by one person, even an unimportant one, may lead the excluded individual to question his or her ability to be included by more important people [38].

Being ostracized is a negative event that will both reduce positive affect and increase negative affect. Research by Leary and several co-authors (Leary et al. 1995) used sociometer theory to redefine self-esteem as “a mechanism by which one assessed one’s inclusionary status” (Williams, 2009, [38,39] (p. 278). The present study argues ostracism (social exclusion) caused by the act of phubbing indirectly and negatively impacts reported levels of anxiety and depression. Williams (2009) concludes that extant research supports the proposed negative outcomes of being ostracized, socially excluded, or rejected [39].

David and Roberts (2017) conducted an experiment to test the model depicted in Figure 1. One hundred and eighty US adults (41% male) were recruited from Amazon’s Mechanical Turk to participate in their study. Subjects were randomly assigned to either a treatment (phubbing) or control condition. Subjects in the treatment condition were asked to read a news article that addressed the issue of being snubbed by others using their smartphones instead of paying attention to the people they are with. The news clip was pre-tested, and it was found that it activated clearly articulated thoughts about the amount of time others were distracted by phones while in their presence.

In David and Roberts’ (2017) main study, subjects were asked to report how often they felt ignored, rejected, or left out while in the presence of others [18]. Subjects also completed scales that measured their need for interpersonal attention, social media use, and psychological well-being (stress and depression). Results of the David and Roberts (2017) experiment found support for the sequential mediation model shown in Figure 1 [18]. Phubbing was found to have a significant indirect positive effect on social media intensity. Additional analyses found that the effects of phubbing extend beyond social media use to heightened levels of reported anxiety and depression. Using a 4-item measure of depression and anxiety developed by Kroneke and colleagues (2009) [40], phubbing was found to be positively associated with both self-reported levels of anxiety and depression. Related research has found that the causal flow is from social media use to well-being, not vice versa ([41,42,43,44] Trumholt, 2016).

The research presented herein expands the generalizability of David and Roberts’ (2017) [18] results by using a phubbing measure (instead of a manipulation of phubbing) to test the relationships uncovered in their sequential mediation model (See Figure 1). We extend David and Roberts’ (2017) [18] research by testing the following research questions: is a measure of perceived phubbing positively associated with feelings of social exclusion and an increased need for attention? Is this increased need for attention associated with heavier social media use? Is a heavier reliance on social media positively associated with anxiety and depression as found by David and Roberts (2017) [18] and others [22,42,43,45,46]? Overall, our model tests whether these downstream effects of phubbing as found in David and Roberts (2017) [18] exist only for particular situational experiences of phubbing (as operationalized through a manipulation as in David and Roberts’ research), or whether these effects also emerge from a general perception of being phubbed by others in day-to-day life. Given that being socially connected is an important precursor of well-being [47], answers to the above questions are essential to our well-being in an increasingly “connected” world.

Next, we present the results of two separate studies. The first study creates and tests the psychometric properties of a scale developed to measure perceived phubbing. A second study tests the model as depicted in Figure 1 as well as provides a second test of the properties of the perceived phubbing measure developed in Study 1. As in David and Roberts (2017) [18], we use a sequential mediation model to test the relationships depicted in Figure 1. However, we extend their research by measuring, rather than manipulating phubbing.

## 4. Study 1

Study 1 presents and tests a new measure of phubbing, defined as one’s perception of being phone snubbed by others while in their company. Specifically, we developed a general measure of phubbing based on an established 9-item measure of partner phubbing [2]. The Roberts and David (2016) measure has been shown to be both a valid and reliable measure of the extent to which an individual is phubbed by his or her relationship partner [2,25,26,27,28]. Thus, we carefully adapted each item of the Roberts and David (2016) measure of partner phubbing to create nine items which would assess general phubbing, rather than partner phubbing (e.g., “My partner glances at his/her cell phone when talking to me” was adapted to “People who I spend time with often glance at their cellphone when talking to me.”) [2]. Full details of the scale are provided in the Appendix.

### 4.1. Sample, Procedure and Measures

A total of 258 (59% female, *M*_age_ = 20, Range_age_ = 18–25) participants completed the measurement validation study. The racial/ethnic breakdown of participants was as follows: 79% white/Caucasian, 7% Latino/Hispanic, 6% Asian/Pacific Islander, 4% black/African American, 1% Native American, and the remaining 3% indicated “other.” Participants were undergraduate students at a large US university; they received course credit for participating in the study.

After being invited into a computer lab and randomly seated at individual computers, participants began the study. First, participants responded to the 9 items that were designed to measure perceived phubbing. Response categories ranged from “never” (1) to “sometimes” (3) to “all of the time” (5).

Next, participants were asked to respond to items from three separate scales. These scales were chosen (one was created) because they are conceptually similar to perceived phubbing and as such, provide a stringent test of the phubbing scale’s discriminant and convergent validity. First, participants responded to a 10-item involvement measure [48] which assessed one’s perceptions of others’ involvement with their cell phones (α = 0.93). Specifically, participants were told that we were interested in understanding how involved they think people who they spend time with are with their cellphones. Participants responded to 10 bipolar items to indicate how they think people who they spend time with perceive their cellphones; example bipolar items included “unimportant—important, boring-interesting, worthless-valuable, not needed-needed.” Second, participants responded to a 5-item measure created for the present study to assess individuals’ perceptions that others are addicted to their cell phones (α = 0.83). Participants responded to this scale by indicating how often they think people that they hang around exhibit various behaviors, including for example, “Try to hide from others how much time they spend on their cell phone,” and “Feel anxious if they have not checked their phone or messages for some time.” Third, participants responded to the 9-item partner phubbing measure (α = 0.91) [2] on which our general phubbing measure was based.

To assess the first link of the model depicted in Figure 1, we included a measure of social exclusion. This is an important test of the phubbing scale’s predictive validity. Specifically, feeling social exclusion during time spent with others (α = 0.90) was assessed using the same scale used by David and Roberts (2017) [18] which asks participants to indicate the extent to which (on a 5-point scale ranging from “not at all” to “very much”), when spending time with other people, they experience feelings of being ignored, left out, and rejected [18]. The order of study measures was randomized to account for any order effects, and each measure was separated by a unique distracting task, such that participants completed several ostensibly unrelated studies [49].

### 4.2. Results

An exploratory factor analysis (EFA) was conducted on the phubbing scale using principal components extraction [50]. The data were well suited for factor analyses, as indicated by the KMO statistic (0.92) and the Bartlett’s test of sphericity (*X*^2^ = 1225.80, *p* < 0.01). The phubbing measure exhibited a factor structure consistent with the hypothesized one factor, and all items loaded strongly (>0.6) onto the single factor. Therefore, and to test discriminant validity of the measure, we next ran a series of confirmatory factor analyses (CFAs) on the scale using AMOS 21.0. Specifically, a CFA was conducted with the 9-item phubbing construct and each of the following: the 10-item involvement construct, the 9-item partner phubbing construct, and the 5-item cell phone addiction measure. Evidence of convergent and discriminant validity was established in each CFA, as all items loaded strongly and significantly on their respective factors and the average variance extracted for each latent variable exceeded 0.50 and exceeded the respective squared correlation between variables [51].

Next, we tested the initial prediction in our conceptual model that perceptions of being phubbed are associated with greater feelings of social exclusion. In support of our hypothesis, perceived phubbing has a significant and positive correlation with feelings of social exclusion (*r* = 0.24, *p* < 0.01). In an effort to further examine the unique predictive ability of perceived phubbing over more generalized measures related to phone use, we next examined the correlations between social exclusion and the measures of involvement and perceived addiction used in the previous tests of discriminant and convergent validity. The correlation coefficients are provided in Table 1. The results show that social exclusion is not significantly correlated with cell phone involvement among others (*r* = –0.08, *p* > 0.10) nor perceptions of others being addicted to their cell phones (*r* = –0.13, *p* > 0.10), thus providing additional evidence of the importance of studying phubbing.

## 5. Study 2

Study 2 was designed to test the sequential mediation model shown in Figure 1 and previously examined in research by David and Roberts (2017) [18]. Thus, the purpose of Study 2 was twofold. Specifically, Study 2 sought to extend research by David and Roberts (2017) [18] by using a measure, rather than manipulation, of phubbing, and examining whether similar effects can be observed for a self-reported measure of perceptions of being phubbed. In addition, Study 2 provided an additional test of the nomological and predictive ability of the proposed nine-item measure of perceived phubbing.

### 5.1. Sample, Procedure, and Measures

One hundred and fifty-seven US adults (46% male, *M*_age_ = 39, Range_age_ = 18–71) from Amazon’s Mechanical Turk participated in Study 2. Sample characteristics and demographics are summarized in Table 2.

Participants were introduced to the study and told that their responses would remain completely confidential. Perceived phubbing (α = 0.94) and feelings of social exclusion (α = 0.90) were assessed using the same measures as in Study 1. Participants’ need for attention (α = 0.91) was assessed using Hill’s (1987) 6-item measure of need for interpersonal attention which has been used previously in related research [52], including in the David and Roberts (2017) study [18]. Participants responded to the 6 statements using a 5-point scale (1 = not at all true, 5 = completely true); example items include “I mainly like to be around others who think I am an important, exciting person,” and “I like to be around people when I can be the center of attention.” Intensity of social media use (α = 0.91) was assessed using the same measure as in David and Roberts’ (2017) research [18], which was based on the Ellison et al. (2007) 6-item measure of Facebook intensity [53]. Participants responded to the 6 items using a 5-point Likert scale; example items include “Social media is part of my everyday activity” and “I would be sorry if social media sites shut down.” Each study measure was separated by a short distracting task. At the end of the study, we assessed participants’ anxiety and depression levels (α = 0.93) using the 4-item measure of depression and anxiety (PHQ-4) developed by Kroenke and colleagues (2009) and used in David and Roberts’ (2017) study [18,40]. Finally, participants responded to demographics, as well as a 9-item measure of self-esteem which would be included as a covariate (α = 0.93) [54].

### 5.2. Results

An exploratory factor analysis was conducted for the 9-item phubbing measure. The data were well suited for factor analyses as indicated by the KMO statistic (0.94) and Bartlett’s test of sphericity (*X*^2^ = 1119.30, *p* < 0.01). Principal components extraction and varimax rotation were used to interpret the factor loadings [49]. The phubbing measure exhibited a factor structure consistent with the hypothesized one factor measure, as items loaded onto one factor, which explained 69% of the variance. In addition, a CFA on the 9-item measure using AMOS 21.0 (*X*^2^ = 69.34, *df* = 27, n = 163; CFI = 0.96; IFI = 0.96; TLI = 0.95), revealed a construct reliability estimate of 0.94 and an average variance extracted of 0.65, thus showing evidence of the measure’s reliability and convergent validity [51].

Means, standard deviations, and correlations for the study measures are reported in Table 3. Since the data for all measures were obtained from the same source, common method variance could bias the results [55]. As such, we performed the Lindell and Whitney (2001) marker variable procedure [56]. Specifically, two items that were expected to be theoretically unrelated to the measures used in the study were imbedded in the questionnaire. The correlations between the marker variable items and each of the study measures were non-significant, small, and close to zero. Therefore, it is unlikely that common method bias affected the results [56,57].

The Preacher and Hayes (2008a) [58] PROCESS macro for SPSS was used to test our predictions and conceptual model shown in Figure 1. This method uses an ordinary-least-squares path analysis to estimate model coefficients and to assess the indirect and/or direct effects of perceived phubbing [59,60]. The PROCESS models use a bootstrapping procedure (n = 5000), which does not rely on any assumptions about the normality of the sampling distribution, to calculate the bias-corrected 95% confidence intervals associated with the statistical significance of the indirect effects [59,60,61].

To test the predicted serial mediation, also referred to as multiple-step, or sequential mediation, the PROCESS Model 6 was conducted [58,59]. This model first tests the relationship between perceived phubbing and participants’ feelings of being socially excluded when spending time with others in person. The results (*F*_(1, 158)_ = 27.41, *p* < 0.01, *R*^2^ = 0.15) indicate that perceived phubbing has a significant relationship with feelings of social exclusion (*β* = 0.46, *p* < 0.01). Second, the model tests whether perceived phubbing and feelings of exclusion during time spent in person with others have a direct relationship with one’s need for attention. The results (*F*_(2, 157)_ = 5.64, *p* < 0.01, *R*^2^ = 0.07) indicate that feelings of exclusion have a significant relationship with need for attention (*β* = 0.28, *p* < 0.01), but the relationship between perceived phubbing and need for attention is non-significant. Third, the model tests the relationship that perceived phubbing, feelings of being excluded during time with others in person and need for attention have with intensity of social media use. The results (*F*_(3, 156)_ = 6.48, *p* < 0.01, *R*^2^ = 0.11) show a significant relationship between both need for attention (*β* = 0.43, *p* < 0.05) and perceived phubbing (*β* = 0.64, *p* < 0.05) and the outcome variable of social media intensity, but a non-significant direct effect of feeling excluded.

Importantly, the results show support for sequential mediation, such that perceived phubbing has a significant indirect relationship with social media intensity via feelings of being excluded during in-person settings and the need for attention (*β* = 0.06, SE = 0.03, 95% CI = 0.01, 0.14). Of note, additional analyses were conducted where self-esteem was included as a control variable. The results did not differ depending on whether self-esteem was included as a control, thus demonstrating that the effects of perceived phubbing are not subsumed or even impacted by a general measure of individual’s self-esteem.

In addition, analyses were conducted to examine possible negative psychological outcomes of the effects predicted and supported in the results above. Specifically, the PROCESS Model 6 [58] was used to test a serial mediation model in which perceived phubbing has a relationship with not only to feelings of exclusion, need for attention, and social media intensity, but also with anxiety and depression. Consistent with the findings by David and Roberts (2017) [18], the results (*F*_(4, 152)_ = 11.26, *p* < 0.01, *R*^2^ = 0.23) showed support for sequential mediation, such that perceived phubbing has a significant and positive indirect relationship with anxiety and depression via feelings of being excluded, need for attention, and then social media intensity.

## 6. Discussion

Because of the attention-grabbing ability of our smartphones, phubbing has become the new norm. Research has shown that the more we are phubbed, the more likely we are to phub others [12,62]. It is a vicious cycle of phubbing others and being phubbed. Across two studies, the present research showed that the perceived phubbing construct and its measurement instrument can significantly further our understanding of the use of cell phones and its effects on feelings of social exclusion. Overall, the results provide support for the predictions that phubbing is positively associated with individuals’ feelings of being excluded during time spent with others in person, and these feelings are positively associated with individuals’ need for attention, which subsequently is positively associated with individuals’ social media intensity. In addition, Study 2 tested an alternative explanation regarding the relationship between perceived phubbing and more general self-esteem. The results demonstrate that the effects of perceived phubbing are not subsumed or even impacted by a general measure of individual’s self-esteem. Further, Study 2 showed that phubbing is indirectly associated with individual well-being, such that those who report higher levels of perceived phubbing also reported higher levels of anxiety and depression.

The attention-grabbing ability of smartphones would only be an interesting story if not for phubbing’s impact on our well-being. As found in David and Roberts (2017) [18] and the present research, the perception of being phubbed is associated with feelings of exclusion and one’s need for attention, which, in turn, are associated with higher levels of social media use. Ultimately, such heightened social media use was found to have negative consequences.

The outcomes of perceived phubbing find theoretical support in both the social exclusion literature and Kardefelt-Winther’s (2014) [35] model of compensatory internet use. Similarly, the results are consistent with an attachment-oriented psychodynamic framework [63,64,65]. Importantly, the lack of a significant direct relationship between perceived phubbing and social exclusion and social media intensity (as well as between social exclusion and social media intensity) in our studies as well as in David and Roberts (2017) [18] study may hint at an interesting theoretical issue related to the importance of understanding the key mechanisms driving social media intensity. As explained by Zhao et al. 2010 and Preacher and Hayes (2008) [58,61,66], the lack of a direct effect between an IV and DV in a mediation model may hint at the omission of one or more mediators. Although the results presented herein found significant indirect effects of perceived phubbing and feelings of social exclusion on social media intensity, the direct effects of these two variables were not significant in predicting social media use. These findings provide further evidence to suggest that a compensatory model underlies the relationship that perceived phubbing and social exclusion have with social media intensity. It appears that experiencing negative life events, such as being phubbed, motivates people to go online (to social media in the present study and David and Roberts, 2017) to mitigate negative feelings caused by the negative life event. That people may turn to social media eschewing face-to-face interactions is noteworthy and potentially troubling. This recently identified tendency to seek social connection online instead of through face-to-face interactions may lead to negative psychological outcomes as demonstrated in the present study, David and Roberts’ (2017) study [18], and a variety of other research studies which have found that social media use is associated with a variety of negative outcomes (c.f., [20]).

The present research found that increased social media intensity is positively associated with anxiety and depression. Similar research has shown that increased social media use leads to interrupted sleep, as well as increases in anxiety, depression and risk factors associated with teen suicide [20,45,46,66,67,68]. Several studies suggest the causal flow is from social media to decrements in well-being, not vice versa [41,42,43,44]. Given the paucity of research on how the common act of phubbing impacts our social relationships and personal well-being, this study makes several important contributions to the current literature.

One important contribution of the present study is the development and validation of a new measure of perceived phubbing (phone snubbing). Research in this area cannot adequately progress without a valid (and brief) measure of its focal variable. As discussed earlier in this manuscript, the few extant “phubbing” scales lack important qualities needed to be able to measure phubbing with a brief and valid scale that can be replicated and used in a variety of research settings.

The scale developed in the present research avoids such problems. Specifically, the scale was found to be unidimensional, reliable (α = 0.94), and possessed convergent and discriminant validity in CFAs with measures of partner phubbing, others’ cellphone involvement, and others’ cellphone addiction. Given the context-specific domain of partner phubbing, we expected, and found, that it is distinct from the new measure of perceived phubbing created in the present study. Overall, the scale presented in the present research offers a parsimonious (9 items), valid, and reliable tool for assessing general phubbing as perceived by any given individual.

It is important to note that the present perceived phubbing scale is a self-report measure of perceived phubbing. Self-report measures have certain advantages but may also suffer from social desirability bias or other potential response sets, improper recall of the events in question (introspective ability of respondent), misunderstanding of questions by respondents, and varying interpretation of rating scales by respondents. It is important for researchers to be mindful of the nuanced conclusions derived from self-report data. The present Study 2 results, however, tend to support that the perceived phubbing scale operates in a manner similar to the manipulation of phubbing as conducted in David and Roberts (2017) [18].

Relatedly, a second important contribution of the present study is its testing of the David and Roberts (2017) phubbing model [18]. The creation of a valid measure of phubbing extends David and Roberts’ (2017) study of the impact of phubbing on social media use and anxiety and depression [18]. Recall, that both David and Roberts (2017) [18], and Study 2 of the present research, found support for the same proposed model of the effects of phubbing; David and Roberts (2017) [18] manipulated phubbing to examine its impact on feelings of social exclusion, social media use, and anxiety and depression, while the present study examined the same relationships but using the new measure of perceived phubbing. Taken together, the two studies show that people turn to social media to compensate for a need for attention spurred by feeling excluded as a result of being phubbed. It should be noted that individuals can deal with feelings of exclusion or ostracism in various ways, such as by engaging in prayer, seeking self-affirmation, or distraction [29], and future research should test moderators on the effectiveness of differing interventions aimed fostering well-being among individuals faced with negative life-events such as being phubbed.

Additionally, both David and Roberts (2017) [18] and the present study provide evidence that heightened social media use is associated with negative psychological consequences. The present study found that perceived phubbing led to higher levels of anxiety and depression through its impact on social media use. This is consistent with a small body of research which finds that it is social media use that leads to lower well-being, not vice versa [41,43]. This relationship could be explained by how people use social media. As originally designed, its intended purpose was to allow people to connect with friends and family. Research, however, suggests that people are not using social media to interact with others. Instead of interacting with others, much of the time spent on social media can be described as “creeping” or “lurking”—viewing others’ pictures and posts without interacting with anyone [24,47]. This type of social media use may be responsible for its negative impact on well-being [42,43,44,45,46].

The increased use of social media may also be eroding our ability to communicate face to face. Spending increasingly less time in face-to-face conversation means that teens are not developing needed social skills [68,69,70,71]. For adults, these important social skills are eroding. As humans, we are keenly attuned to social information. To be able to understand and relate to others is both a complex task and a vitally important ability. It is all very primordial. Our social skills are closely tied to our survival and ability to procreate. Mastering these all-important social skills takes practice. Observing subtle non-verbal skills such as eye movement, posture, vocal intonations, touch, and response to ambient environmental cues allows us to better understand the intentions of our conversation partners [68]. Will the attention-grabbing ability of the modern smartphone continue to interrupt our face-to-face conversations and erode these very skills that are so important to building and maintaining healthy relationships?

## 7. Limitations and Future Research Directions

Although the present research is the first to utilize a parsimonious, valid, and reliable measure of perceived phubbing and to investigate its downstream influence on social media use and anxiety and depression, its results must be tempered by certain limitations. First, although we used two separate samples of US college students and adults, future research will benefit from collecting data from samples of adolescents where this behavior is common and larger, random samples. It would be helpful to have a deeper understanding of the type of people who are most likely to phub or be phubbed. An earlier study suggests females are more likely to phub others [18]. This is consistent with research that has found that smartphone addiction leads to phubbing behavior [12], and that females are more likely to be addicted to their smartphones [2].

A second potential limitation of the present research is its correlational nature. The present study did, however, broaden the generalizability of the David and Roberts (2017) [18] phubbing study which manipulated phubbing to investigate its impact on social media use and well-being. Although several other studies have found that heightened social media use leads to lower well-being [41,42,43], additional research is needed to further address the direction of causal flow.

Third, although the present research has developed an arguably valid measure of perceived phubbing, it will take additional studies across a variety of situations to provide further evidence of its usefulness in studying phubbing behavior. A valid measure of the increasingly common behavior of phubbing is needed, including a measure from the phubber’s perspective, as this could be used to develop a profile of the typical phubber, as well as to identify antecedents and consequences of phubbing.

An additional limitation and area for further research would be the explanation used to describe the positive relationship between social media use and anxiety and depression. On the surface, it appears, as originally designed, that social media use should strengthen social relationships, and hence, improve well-being. In fact, Elhai et al. (2017) [72] found that social smartphone use (social media and messaging) led to lower levels of self-reported anxiety compared to non-social use (news and entertainment) of one’s smartphone. Like many technologies, however, users are accessing social media, not to interact, but to “stalk,” “creep,” or “lurk” on others [73]. The idealized depiction of others’ lives on social media suggests that when we are comparing ourselves to others via social media, we often compare unfavorably leading to lower well-being. Future research needs to take a closer look at how social media is being used.

It is possible that social media’s negative impact on well-being may be explained as a matter of opportunity costs. The more one uses social media, the less he or she has face-to-face interactions which have been shown to increase well-being [42]. An important question to be addressed is, “Are we losing both the ability and desire to interact face to face?” If so, this trend of engaging in distracted interactions with others or avoiding face-to-face conversations altogether will continue to contribute to an increasing sense of social exclusion and its attendant negative psychological outcomes of anxiety and depression.

## 8. Conclusions

Living in a highly digitized environment has its advantages and disadvantages. One such disadvantage was the subject of the present research—phone snubbing (phubbing). The modern smartphone is ubiquitous and omnipresent. A growing body of research has shown that smartphones may hold sway even during face-to-face conversations and everyday social situations. This same research shows that such behavior can undermine relationship quality in romantic partners and perceived conversation quality among conversation partners. The present study made two important contributions to the “phubbing” literature. First, we developed a parsimonious (9-item), valid and reliable measure of perceived phubbing. Extant phubbing measures lack important psychometric qualities that would make their use in a variety of research settings problematic. A second important contribution of the present study is that we used this newly created perceived phubbing scale to test and extend David and Roberts’ (2017) [18] sequential mediation model of phubbing. Social exclusion theory and Kardefelt-Winthers’ (2014) [34] model of compensatory internet use provide strong theoretical support for the David and Roberts (2017) model. When people experience negative life events they turn to social media (or the internet) in an attempt to alleviate the feelings of exclusion engendered by being phubbed. The present study’s results find that increasing one’s social media use as a means of coping with feelings of social exclusion is associated with higher levels of reported anxiety and depression. Given the seemingly non-stop digitization of our world, research that investigates how technology use influences human well-being is critical.

## Figures and Tables

**Table 1:** Correlation Coefficients.

	Perceived Phubbing	Social Exclusion	Perceived Cellphone Involvement of Others
Perceived Phubbing	–		
Social Exclusion	0.24 *	–	
Perceived Cellphone Involvement of Others	0.20 *	–0.08	–
Perceived Cellphone Addiction of Others	0.33 *	–0.13	0.27 *

Note: * *p* < 0.05.

**Table 2:** Demographic Profile of the Sample.

Sample (n = 157)	Percentage
Gender Female Male	54 46
Education Less than High School High School/GED Some College 2 Year College Degree 4 Year College Degree Master’s Degree Doctorate Degree	1 9 27 10 38 13 2
Relationship Status Single Married Divorced Widowed	47 41 10 2
Ethnicity White/Caucasian Asian/Pacific Islander Hispanic African American Native American Other	76 8 8 6 1 1
Income Less than $20,000 $20,000 to $44,999 $45,000 to $49,999 $50,000 to $59,999 $60,000 to $69,999 Over $70,000	19 29 13 9 6 24

**Table 3:** Means, Standard Deviations, and Correlation Coefficients.

	*M*	SD	Marker Variable 1	Marker Variable 2	Perceived Phubbing	Feelings of Exclusion	Need for Attention	Social Media Intensity
Marker Variable 1	2.90	1.38	--					
Marker Variable 2	3.94	0.72	−0.10	--				
Perceived Phubbing	3.39	0.84	0.03	−0.03	--			
Feelings of Exclusion	2.31	0.99	0.11	−0.05	0.39 *	--		
Need for Attention	2.25	0.97	0.04	−0.03	0.04	0.25 *	--	
Social Media Intensity	4.73	1.84	0.02	−0.07	0.25 *	0.06	0.21 *	--

Note: * *p* < 0.05.

## Appendix A

Phubbing Measure *

The first set of questions asks about how often people who you spend time with (i.e., friends, neighbors, family, etc.) exhibit certain behaviors.

Please use the scale provided to indicate how frequently you experience each of the following behaviors while spending time with other people.

1. During a typical mealtime that I spend with people, they pull out and check their cellphone.
2. When their cellphone rings or beeps, they pull it out even if we are in the middle of a conversation.
3. During leisure time that I spend together with others, the person/people use their cellphone.
4. People who I spend time with often glance at their cellphone when talking to me.
5. When I spend time with people, they keep their cellphone where they can see it.
6. People use their cellphones when we are talking in person.
7. People never keep their cellphones in their hand when they’re with me. (R)
8. When I am out with others, they use their cellphone at some point during our time together.
9. If there is a lull in my conversation with others, they will check their cellphone.

* Adapted from Roberts and David (2016) [2] 9-item measure of Partner Phubbing in which items 1, 4, and 6 were adapted from McDaniel and Coyne’s (2016) TILES scale [23]. Response categories ranged from “Never” (1) to “Sometimes” (3) to “All of the Time” (5).
